# Assessment of Eating Attitudes and Body Image Among 17–20-Year-Olds Engaged in Regular Sports Activity

**DOI:** 10.3390/nu17213482

**Published:** 2025-11-05

**Authors:** Martyna Biedroń, Sylwia Jaruga-Sękowska, Martyna Kłoda, Wiktoria Staśkiewicz-Bartecka, Joanna Woźniak-Holecka

**Affiliations:** 1 Department of Health Promotion, Faculty of Public Health in Bytom, Medical University of Silesia in Katowice, Ul. Piekarska 18, 41-902 Bytom, Polandjwozniak@sum.edu.pl (J.W.-H.); 2 Department of Physical Activity and Health Promotion, Academy of Physical Education in Katowice, Mikolowska 72a, 40-065 Katowice, Poland; 3 Department of Food Technology and Quality Evaluation, Department of Dietetics, Faculty of Public Health in Bytom, Medical University of Silesia in Katowice, ul. Jordana 19, 41-808 Zabrze, Poland; wstaskiewicz@sum.edu.pl

**Keywords:** eating disorders, body esteem scale, athletes, adolescence, BMI, sports psychology

## Abstract

Background/Objectives: Eating disorders (EDs) and body image disturbances are increasingly recognized as important health issues among young athletes. Sports participation may both support healthy development and simultaneously increase vulnerability to disordered eating due to performance pressures and cultural ideals. The aim of this study was to assess the risk of eating disorders and body image among 17–20-year-old athletes. Methods: The study included 428 participants (215 women and 213 men) actively engaged in sports. Standardized psychometric tools were applied, including the Eating Attitudes Test (EAT-26) and the Body Esteem Scale (BES). Statistical analyses examined differences across gender, BMI categories, and sports disciplines, as well as predictors of ED risk. Results: The analysis showed that 32.9% (*n* = 141; 95% CI: 28.3–37.8%) of respondents were at risk of developing eating disorders, with women being significantly more vulnerable than men (*p* < 0.001; V = 0.27). Underweight athletes demonstrated a higher risk compared with those of normal weight (OR = 2.86, 95% CI: 1.48–5.55, *p* < 0.001). The type of sport was also associated with risk (*p* < 0.001, V = 0.323); the highest prevalence of ED risk occurred among dancers (48.1%) and swimmers (38.9%). Body esteem differed markedly between groups: participants at risk scored lower in Weight Control (*p* < 0.001, Cohen’s d = 0.94) and Physical Attractiveness (*p* = 0.072) but higher in Physical Condition (*p* < 0.001). Regression analyses indicated that gender (β = −3.35, *p* < 0.001) and Body Esteem—Weight Control (β = −0.45, *p* < 0.001) were the strongest predictors of EAT-26 scores. Conclusions: The findings confirm the multidimensional nature of eating disorder risk among young athletes, highlighting the role of body image imbalance and gender differences. Early screening, preventive interventions, and multidisciplinary support are essential to protect both the physical and mental health of young athletes. Future research should include objective physiological measures and broader samples to improve generalizability.

## 1. Introduction

Issues related to body image and eating disorders are an important area of interest in contemporary research in developmental psychology, public health, and sports sociology. In an era dominated by visual culture and the growing importance of socially promoted ideals of slimness and physical attractiveness, young people in a crucial phase of psychological development experience intense pressure related to their appearance. Research confirms that eating disorders are increasingly emerging as a consequence of cultural and media factors [1], as well as social conditions that shape an individual’s attitude towards their body and eating habits [2]. The contemporary cultural context is characterised by the coexistence of two dominant discourses: on the one hand, the idea of rational and healthy eating as part of a healthy lifestyle is promoted, while on the other hand, there is persistent normative pressure to be slim. These seemingly contradictory trends result in increased interest in nutrition and a conscious, often controlled approach to food consumption. Although the pursuit of self-control can motivate individuals to improve their health or appearance, it is not always safe or healthy [3,4].

Adolescence constitutes a developmental period particularly sensitive to both psychological and physiological changes. Among individuals actively engaged in sports, there is an increased body awareness, which amplifies the significance of self-perception of appearance. Concurrently, athletic competition imposes elevated expectations regarding both performance outcomes and physical appearance. Epidemiological data concerning the prevalence of eating disorders (EDs) among adolescents and young adults are alarming and underscore the urgent need for further investigation [5,6]. Notably, individuals exhibiting low self-esteem and high levels of perfectionism—especially within the athlete population—demonstrate a heightened incidence of ED symptomatology [7].

Within the context of physical activity, it is important to acknowledge that although regular sports participation confers numerous health benefits, intense competitive involvement may be associated with an elevated risk of developing eating disorders, particularly among females. These disorders exert deleterious effects on both physical and mental health, diminish overall well-being, and impair athletic performance [8,9]. The pressure inherent in sport is primarily linked to the imperative of maintaining a slender physique, which is commonly regarded as an indicator of success—especially in disciplines where a lean body composition facilitates performance and aligns with the aesthetic criteria imposed by judges and the sporting environment. Moreover, the escalating level of competition intensifies pressure and motivation to engage in maladaptive behaviors aimed at enhancing athletic results or managing stress.

Although discipline and dedication are fundamental to athletic achievement, these traits may engender adverse social consequences, particularly when they transgress broadly accepted societal norms and standards. The prevailing narrative of discipline frequently manifests in dietary practices, rendering nutrition and food regulation pivotal not only for physical fitness but also for body image construction [1,10]. Additionally, regular physical activity plays a critical role in shaping body image among youth, although this effect is subject to modulation by factors such as gender and the specific characteristics of the sport practiced [11,12].

The present study aims to conduct a comprehensive cross-sectional assessment of the prevalence of eating disorder (ED) risk and body image perception among individuals aged 17–20 years who actively participate in sports. The primary objective is to determine the overall proportion of young athletes at risk of developing EDs within this population. Additionally, the study explores secondary objectives, including the identification of factors that may influence this risk, such as gender, age, type of sport practiced, perceived stress, and the level of pressure experienced from coaches. It is hypothesized that higher risk levels may occur among females and among athletes engaged in disciplines characterized by greater aesthetic or weight-related demands, where both biological predispositions and socio-cultural factors contribute to vulnerability. This investigation seeks to provide robust empirical evidence to enhance understanding of the mechanisms underlying ED risk in young athletes and to inform the development of targeted preventive and therapeutic interventions within the sports context.

## 2. Materials and Methods

### 2.1. Procedure of the Study

The present study was conducted from December 2024 to July 2025 and involved participants aged 17–20 years. Data collection employed the computer-assisted web interviewing (CAWI) technique through a standardized online questionnaire. This method is widely recognized in psychological research as a reliable and valid instrument for collecting self-reported data, particularly appropriate for adolescent and young adult populations due to its accessibility and user-friendliness. Prior to participation, respondents were provided with comprehensive information about the study objectives, assured of full anonymity, and given detailed instructions on questionnaire completion. Participation was voluntary and based on informed consent, with individuals explicitly informed of their right to withdraw at any time without penalty or obligation to justify their decision. The study was conducted in strict accordance with the ethical principles outlined in the Declaration of Helsinki by the World Medical Association. Ethical approval was obtained from the Bioethics Committee of the Medical University of Silesia in Katowice (No. BNW/NWN/0052/KB254/23, dated 20 November 2023). Thus, the research adhered rigorously to both international and national ethical standards.

### 2.2. Participants

The study employed a defined sampling procedure designed to ensure high precision and reliability of the empirical data obtained. The sample size calculation was based on statistical assumptions, including a 95% confidence level, a 5% margin of error (0.05), and an estimated proportion of 0.5. The sample size was determined using a standard formula for estimating population proportions. This approach ensured statistical stability of the estimates and permitted drawing conclusions with a high degree of accuracy for the target population.

**Figure 1:**
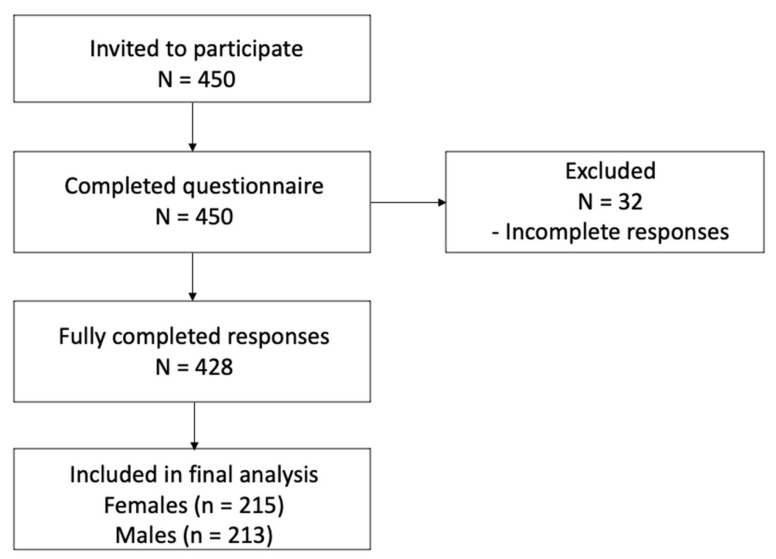
Diagram showing the recruitment process and final sample size.

An online questionnaire was sent to individuals aged 17–20 who regularly participate in sports and attend secondary schools, and sports clubs in the Silesian Province (Poland). The sample was purposive, including individuals who actively participated in at least 3 training sessions per week (≥150 min in total) for at least six months. The participants were a group of amateur athletes who regularly participated in training and competitions at a recreational or school level. A total of 450 respondents participated in the study. After verifying the accuracy and completeness of the data, 428 fully completed questionnaires were included in the final analysis, and 32 were excluded due to missing answers (Figure 1). The inclusion criteria were: (1) voluntary participation with complete answers to the questionnaire questions, (2) age between 17 and 20 years, and (3) self-reported regular participation in sports. Exclusion criteria included diagnosed mental or neurological disorders potentially affecting the reliability of responses, age outside the 17–20 range, and lack of physical activity.

### 2.3. Survey Tools

The study employed a multi-part questionnaire that included both original items and standardized measurement instruments. The introductory section collected sociodemographic and anthropometric data, such as age, height, body weight, and the presence of chronic diseases (e.g., mental health disorders such as depression or eating disorders). It also contained questions related to physical activity, including the type of sport practiced, frequency of exercise, perceived pressure from a coach, and whether physical activity was perceived as a means of coping with stress. Subsequent sections incorporated validated research tools with established reliability and validity: the Eating Attitudes Test-26 (EAT-26), used to assess the risk of eating disorders, and the Body Esteem Scale (BES), used to evaluate satisfaction with various aspects of body image. The inclusion of these instruments enabled a comprehensive and multidimensional analysis of psychological factors and eating behavior patterns among the study participants.

#### 2.3.1. Body Esteem Scale BES

This study employed the Body Esteem Scale (BES) questionnaire, originally developed by Franzoi and Shields [13] and subsequently adapted into Polish by M. Lipowska and M. Lipowski [14]. The BES is a standardized psychometric instrument designed to assess individuals’ subjective perceptions of their own bodies. The questionnaire comprises 35 items, organized into three subscales, with the subscale structure differing according to the respondent’s gender. For male participants, the subscales included physical attractiveness, body strength, and physical condition, whereas for female participants, the subscales encompassed sexual attractiveness, weight control, and physical condition. For comparative purposes, gender-specific subscales were matched conceptually: the Physical Attractiveness subscale for men corresponded to Sexual Attractiveness for women, while Weight Control (women) and Body Strength (men) were analyzed jointly as indicators of body regulation. The Physical Condition subscale was common to both genders. Respondents rated each item using a five-point Likert scale, where 1 indicated strong negative feelings, 5 indicated strong positive feelings, and 3 represented a neutral attitude. Subscale scores were calculated as the arithmetic mean of the item scores within each respective category. Raw scores were subsequently transformed into sten scores ranging from 1 to 10. Interpretation of these sten scores followed established guidelines: scores between 1 and 3 indicated low body esteem, 4 to 7 indicated average body esteem, and 8 to 10 indicated high body esteem [13,14].

#### 2.3.2. Eating Attitudes Test-26

The Eating Attitudes Test-26 (EAT-26), developed by Garner and Garfinkel, is a standardized diagnostic instrument designed to assess attitudes, beliefs, and behaviors related to eating and nutrition. It is among the most widely utilized screening tools in epidemiological research on the prevalence of eating disorders globally. Empirical evidence supports its high efficacy in identifying individuals at elevated risk of developing eating disorders, particularly within adolescent and student populations [15,16].

The questionnaire comprises three subscales: (1) Dieting, (2) Bulimia and preoccupation with food, and (3) Oral control. Subscale scores are calculated by summing the points assigned to responses within each domain. For the purposes of this study, the total score encompassing all 26 items was analyzed. Responses are rated on a five-point Likert scale, with items 1–25 scored as follows: ‘Always’ = 3 points, ‘Usually’ = 2 points, ‘Often’ = 1 point, and other responses = 0 points. Item 26 employs a reverse scoring system. The total possible score ranges from 0 to 78, with a threshold score of ≥20 indicating increased risk of an eating disorder and warranting referral for specialized diagnostic evaluation. Higher scores correspond to greater symptom severity.

The EAT-26 demonstrates high reliability and robust psychometric properties, making it a valuable tool for both clinical assessment and population-based research. Utilizing a total score as a global index of eating attitudes facilitates interpretation and comparison of results across individuals and groups. Consequently, the EAT-26 is extensively employed in comparative studies and investigations of risk factors for eating disorders, supported by well-established normative data that enable meaningful interpretation across diverse populations.

### 2.4. Statistical Analysis

The collected data were subjected to statistical analysis using Statistica v.13.3 (Stat Soft Poland, Kraków, Poland). Descriptive statistics (means, standard deviations, frequencies, and percentages) were calculated for all variables. Normality of distributions was assessed using the Shapiro–Wilk test. Depending on the type of data, Student’s *t*-test, Mann–Whitney U test, or chi-square test (χ^2^) were used for comparisons between groups. Additionally, effect sizes (Cohen’s d, Cliff’s delta, and Cramér’s V) were calculated to assess the strength of the association. For variables with more than three categories, post hoc comparisons with Bonferroni correction were performed for significant chi-square test results to identify specific differences between groups. Analysis of covariance (ANCOVA) was performed to assess differences in EAT-26 scores between groups of participants engaging in different types of physical activity, controlling for demographic and anthropometric variables. Age, gender, and BMI were included as covariates, and the model also included the interaction effect between type of physical activity and gender. The strength of the relationship between the observed and predicted values of the dependent variable was evaluated using the multiple correlation coefficient (R), while the proportion of variance in EAT-26 explained by the model was assessed using the coefficient of determination (R^2^). The results presented regression coefficients (β) along with 95% confidence intervals and *p*-values, with a significance level of α = 0.05.

## 3. Results

A total of 428 individuals aged 17–20 participated in the study. Women constituted 50.2% (*n* = 215) of the sample, while men accounted for 49.8% (*n* = 213). The average age of the participants was 18.6 years (SD = 1.13) and did not differ significantly between sexes: women were on average 18.7 ± 1.09 years old, and men 18.6 ± 1.19 years old (*p* = 0.561). The mean height for the entire group was 175 cm (SD = 9.92), with women being significantly shorter (168.7 ± 7.07 cm) than men (181.4 ± 8.14 cm) (*p* < 0.001). The average body weight of all participants was 68.5 kg (SD = 13.88). Women weighed on average 59.4 ± 10.03 kg, while men weighed 77.6 ± 10.94 kg, representing a statistically significant difference (*p* < 0.001). The mean body mass index (BMI) for the whole sample was 22.2 kg/m^2^ (SD = 2.99). Women had a significantly lower BMI (20.9 ± 3.24) compared to men (23.5 ± 2.04) (*p* < 0.001) (Table 1).

**Figure 2:**
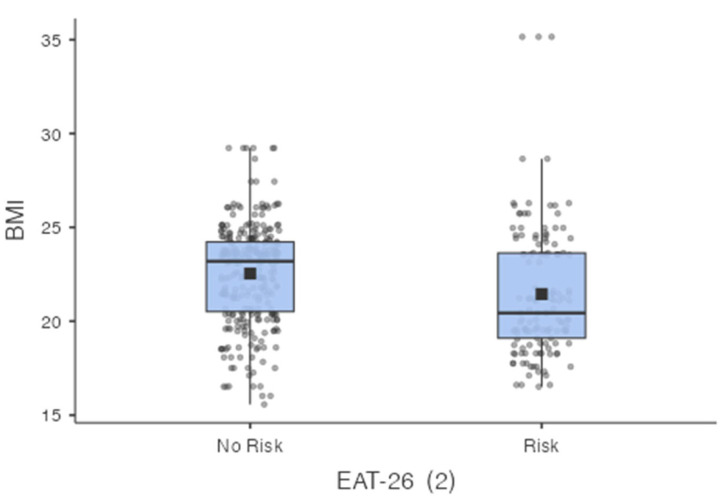
Distribution of BMI values in groups of people without risk (*n* = 287) and at risk (*n* = 141) of eating disorders based on the results of the EAT-26 questionnaire.

Figure 2 presents a comparison of body mass index (BMI) values between respondents classified as having no behaviors indicative of an increased risk of eating disorders (No Risk) and those identified as being at increased risk (Risk), according to the EAT-26 questionnaire. The distribution of BMI values in the No Risk group is slightly shifted towards higher values compared to the Risk group. In the increased-risk group, a greater concentration of BMI values is observed in the lower range of the normal BMI category.

The analysis revealed significant correlations between the type of sport practiced and the risk of eating disorders (*p* < 0.001; V = 0.323). In the group of people at increased risk, swimmers (58.1%) and dancers (27.6%) accounted for a particularly high percentage, while people without risk more often chose the gym (35.0%) or volleyball (23.1%). When broken down by gender, a significant relationship between the type of activity and risk was found in men (*p* = 0.002; V = 0.299), while in women the relationship did not reach statistical significance (*p* = 0.382).

The extent to which physical activity helps to cope with stress and the difficulties of everyday life was also assessed. Overall, most respondents considered training to be an important form of support. This percentage was higher in the group without risk of eating disorders (67.6%) compared to the group at risk (54.6%). The differences were statistically significant in the entire sample (*p* < 0.001; V = 0.238) and among men (*p* < 0.001; V = 0.360), while among women this effect did not reach significance (*p* = 0.096).

The majority of respondents reported experiencing pressure from their coach regarding the assessment of their physique. Among participants without risk of eating disorders, 67.2% declared feeling such pressure, whereas this proportion increased to 82.3% among those at risk. The differences were statistically significant for the total sample (*p* = 0.005; V = 0.158). When analyzed by gender, similar patterns were observed. Among women, 82.7% of those at risk and 57.3% without risk reported feeling pressure from their coach (*p* < 0.001; V = 0.274). Among men, the respective values were 81.4% (risk) and 74.1% (no risk), but this difference did not reach statistical significance (*p* = 0.305; V = 0.106).

Comparing one’s own appearance with that of others with whom the respondents train was a common phenomenon–in the group of people at increased risk of eating disorders, as many as 78% of respondents reported such behaviour, while in the group without risk, the percentage was 62%. Women at increased risk were more likely to admit to comparing their appearance (86.5%) than women without this risk (65.0%). Among men, however, the level of comparison was similar (no risk–60.0%; at risk–58.1%). The differences were statistically significant in the entire sample (*p* = 0.003; V = 0.164) and among women (*p* < 0.001; V = 0.270), but not among men (*p* = 0.933).

Importantly, regular physical activity clearly modified attitudes towards food. Across the entire sample, as many as 94.3% of individuals at increased risk of eating disorders declared that training had changed their approach to food. This result was particularly evident among women (94.3%) and men (93%) at increased risk of eating disorders. The analysis showed significant differences in all comparisons: in the entire sample (*p* < 0.001; V = 0.404), among women (*p* < 0.001; V = 0.386) and among men (*p* < 0.001; V = 0.363) (Table 2).

The analysis of covariance revealed a significant main effect of gender on EAT-26 scores, F = 12.45, *p* < 0.001. Women obtained significantly higher EAT-26 scores compared to men, independent of age, BMI, and type of physical activity. No significant differences were found in EAT-26 scores across types of physical activity (*p* = 0.374), and there was no significant interaction between gender and type of activity (*p* = 0.491) (Table 3). Age and BMI were not significant covariates in the model (*p* > 0.05).

According to EAT-26 scoring, 32.9% (*n* = 141; 95% CI: 28.3–37.8%) of participants were classified as being at risk of eating disorders. Based on the calculated BMI values, 11% (*n* = 47) of respondents in the total study sample were underweight, 76.2% (*n* = 326) had a normal body weight, 12.1% (*n* = 52) were overweight, and 0.7% (*n* = 3) were classified as obese. Among participants at increased risk of eating disorders, 18.4% (*n* = 26) were underweight, 70.2% (*n* = 99) had a normal body weight, 9.2% (*n* = 13) were overweight, and 2.1% (*n* = 3) were obese. In the non-risk group, 7.3% (*n* = 21) were underweight, 79.1% (*n* = 227) had a normal body weight, 13.9% (*n* = 39) were overweight, and no cases of obesity were recorded. Post hoc comparisons with Bonferroni correction showed that participants classified as underweight or obese had significantly higher EAT-26 scores than those with normal body weight (*p* < 0.001).

The analysis of the relationship between the Body Esteem Scale and the risk of eating disorders (EAT-26) revealed several significant patterns. In the Physical Attractiveness subscale, individuals at risk of eating disorders significantly more often reported low levels of body esteem (56.0%) compared to those without risk (33.8%), whereas high scores were more frequent in the no-risk group (33.1% vs. 15.6%; *p* < 0.001). A particularly strong association was observed in the Weight Control dimension (*p* < 0.001; V = 0.322). Almost half of the at-risk respondents (47.5%) reported low levels of body esteem in this area, compared to only 19.2% in the no-risk group. Conversely, high scores were substantially more frequent among individuals without risk (44.3%) than among those at risk (18.4%). In the Physical Condition subscale, the differences were also significant (*p* < 0.001; V = 0.291). Half of the at-risk group (50.4%) obtained high scores in this dimension, in contrast to only 26.8% of respondents without risk. At the same time, low levels were much more common in the no-risk group (42.9%) than in the risk group (15.6%). Detailed data are presented in Table 4.

The mean EAT-26 score for the entire sample was 15.84 ± 12.94 and was significantly higher in women (20.33 ± 13.64) than in men (11.31 ± 10.42; *p* < 0.001). A similar pattern was observed in the Dieting, Bulimia, and Oral Control subscales, with women scoring significantly higher in all cases (all *p* < 0.001).

Analysis of the Body Esteem Scale revealed mean scores of 40.07 ± 8.18 for Physical Attractiveness, 31.17 ± 10.13 for Weight Control, and 40.24 ± 14.26 for Physical Condition. No significant gender differences were observed in the Physical Attractiveness subscale (*p* = 0.072). However, in the Weight Control subscale, men scored significantly higher (35.61 ± 8.11) than women (26.76 ± 10.02; *p* < 0.001). A similar and even more pronounced difference was noted in the Physical Condition subscale, with men scoring substantially higher (50.07 ± 10.72) compared with women (30.51 ± 10.02; *p* < 0.001) (Table 5).

The linear regression model showed a moderate relationship between the independent variables and the EAT-26 test result (R = 0.551), explaining 30.4% of the variance in the dependent variable (R^2^ = 0.304). Among the predictors analysed, the weight control subscale proved to be a significant factor (t = −5.15; *p* < 0.001). Gender was also a statistically significant predictor (t = −2.12; *p* = 0.034)—women scored higher on the EAT-26 test than men.

In addition, body weight category was significantly associated with EAT-26 scores—obese individuals scored higher (*p* < 0.001), while individuals with normal weight scored lower (*p* = 0.023) compared to overweight individuals.

Importantly, participants who swam scored slightly higher on the EAT-26 test compared to those who indicated running as their main form of physical activity (t = 1.94; *p* = 0.05).

In contrast, the results of the BES–Physical Attractiveness (*p* = 0.494) and BES–Physical Fitness (*p* = 0.237) subscales, as well as other types of physical activity, did not show any significant effects (Table 6).

## 4. Discussion

The aim of the study was to assess the risk of eating disorders and body image among 17–20-year-olds who participate in sports. The results of the study are important for both the physical and mental health of young people and have significant implications for doctors, teachers and mental health professionals. The results confirm the multidimensional nature of the risk of eating disorders among young athletes. One manifestation of this risk is an imbalance in body image—those at risk are more likely to have a low sense of attractiveness and control over their weight, while at the same time having a high sense of physical fitness. This observation is consistent with earlier reports emphasising the importance of cultural and social pressure on women, for whom a slim figure is considered an important element of attractiveness and sporting success [17,18].

The results of the EAT-26 questionnaire analysis showed that approximately 32.9% of respondents are at risk of developing eating disorders and should see a specialist for further diagnosis. The results show that gender is an important factor in differentiating the risk of eating disorders–women in the study sample are significantly more at risk of these problems than men.

It is worth noting that significant differences were observed between body mass index (BMI) and increased risk of eating disorders in the study group. The results suggest that being underweight is associated with an increased risk of eating disorders, while overweight and normal-weight athletes are less prone to these disorders.

The results of the study by Mäkinen et al. confirm that body dissatisfaction is strongly associated with attitudes towards body weight and negative eating habits already in adolescence, especially in girls [19]. The results of Borowiec et al. also show that the risk of developing eating disorders is higher in female athletes with low body satisfaction and depending on the type of sport practised, the level of competition and BMI [20]. In turn, Pustivšek et al. point to differences in the risk of ED between athletes and non-athletes—in young athletes, both in female and male groups, abnormal eating patterns are more often associated with low self-esteem [21]. These differences may result from greater socio-cultural pressure to be slim, a stronger link between appearance and self-esteem, and the influence of biological factors such as hormonal fluctuations, which can affect body image and relationship with food [19,22].

Interestingly, the authors’ study showed that underweight individuals were more prone to eating disorders. However, a study by Memon et al. in a group of students showed the opposite result, i.e., overweight individuals are at a higher risk of developing eating disorders compared to underweight individuals. In 18.2% of overweight participants, the result exceeded the threshold value suggesting the presence of eating disorders, while in the group of underweight individuals, this percentage was 15.6% [23]. The difference in the tendency to develop eating disorders observed in the study may be due to the fact that the study by Memon et al. was conducted in the general student population, while in our own study, the study group consisted of people who practised sport. These differences may result from the specific nature of the sports environment, where pressure to maintain a certain body shape and the pursuit of low body weight are often much stronger. Underweight in athletes may be the result of deliberate actions, such as restrictive diets or excessive physical activity, which increase the risk of eating disorders. In addition, personality traits typical of athletes, such as perfectionism and high self-control, may contribute to the development of eating disorders [23,24].

The study revealed a significant correlation between the type of discipline practiced and the risk of eating disorders (*p* < 0.001; V = 0.323). People who practice dance were found to be most susceptible to developing abnormal eating behaviours—almost half of them scored positively on the EAT-26 questionnaire, which indicates a particularly strong link between this activity and the risk of eating disorders. Dance is a discipline that is strongly focused on appearance, physique and body aesthetics. High demands on weight and body proportions can lead to pressure and increased awareness of one’s own appearance, which increases the risk of eating disorders. As demonstrated, among others, in the study by Arcelus et al., dancers are up to three times more likely to develop eating disorders than the general population [25]. In the study by Diogo et al., eating disorders affected nearly one-third of ballet dancers, with a clear predominance of cases among women [26]. Similar results were reported by Douglas et al. when analysing the environment of academic dancers—it was found that 18.75% of the respondents showed symptoms of eating disorders, with women being significantly more at risk [27]. This phenomenon can be explained by the specific nature of the dance environment, where the emphasis on a slim figure, the aesthetics of movement and stage requirements result in strong pressure to maintain a low body weight. Young women who are highly achievement-oriented, competitive and perfectionist often develop what is known as neurotic perfectionism. It is characterised by disproportionately high demands for oneself and one’s environment, and failure to meet these expectations results in lowered self-esteem and increased internal criticism. Such psychological mechanisms can be a significant risk factor for eating disorders, especially in populations such as dancers or aesthetic sports competitors [28].

The group at risk of abnormal eating behaviors also includes people who train in swimming. Although swimming is characterised by moderate social pressure, in competitive environments, athletes are often judged on their body weight and muscle proportions, which may increase the risk of eating problems. In addition, the characteristic outfit that reveals the figure may increase critical assessment of one’s own body. Particularly high risk rates were found among swimmers and dancers, which is consistent with the results of previous studies indicating that sports that emphasise weight and appearance are associated with more severe symptoms of eating disorders [7,9]. Swimming, although perceived by society as involving moderate aesthetic pressure, often generates additional expectations in a competitive environment regarding body weight and muscle proportions, which have a direct impact on athletic performance. Wearing a swimsuit that exposes a significant part of the body increases the exposure of the figure and thus may increase social comparisons and critical assessment of appearance. Similar relationships were noted by Pustivšek, indicating that in a group of swimmers, the risk of eating disorders is associated with both environmental pressure and reduced body satisfaction [21]. Our results are also consistent with the observations of Borowiec et al. who emphasise that in sports with a clear body image component, such as swimming, body dissatisfaction and high aesthetic demands are particularly important risk factors for the development of eating disorders [20]. Gender analysis showed a significant relationship between the type of activity and the risk of eating disorders in women (*p* = 0.002; V = 0.299). In men, however, this relationship did not reach statistical significance (*p* = 0.382).

The results of this study confirm a significant relationship between body image and the severity of eating disorder symptoms. The use of the BES questionnaire in population studies allows for the early detection of individuals with a negative body image, which may require further and in-depth diagnosis and specialist intervention [14]. People with low scores on the body assessment scale were significantly more likely to have elevated scores on the EAT-26 test, suggesting that a negative body image is a risk factor for the development of abnormal eating attitudes. This relationship is consistent with the findings of Sharif-Nia et al. whose study showed that individuals with low scores on the BES test had high scores on the EAT-26 [29]. The results confirm that low body image is a significant predictor of unhealthy eating attitudes. In this context, body image appears to be an important risk factor that should be taken into account in both diagnosis and the planning of preventive and therapeutic measures [24,29,30].

The present study showed that the group of athletes significantly compares themselves to their peers and feels pressure regarding their appearance in their training environment. The results are consistent with those obtained by Shroff et al. and clearly indicate the important role of peer influence in shaping body image, self-esteem and the occurrence of eating disorders in teenage girls. The analysis showed that comparing appearance with peers and internalising the ideal of thinness were strongly associated with body dissatisfaction, symptoms of eating disorders and low self-esteem [31]. A particularly noteworthy finding was the high prevalence of perceived pressure from coaches regarding body image—reported by over 80% of at-risk respondents. This confirms that coach-related expectations constitute a significant psychosocial factor that may contribute to disordered eating behaviors among young athletes. The study conducted by Stoyel et al. also proves that athletes are highly susceptible to social pressure, which comes from both their immediate environment and wider social circles. This type of pressure promotes a stronger identification of physical appearance with athletic competence, especially in situations where individuals compare their physique with that of athletes whose achievements serve as a model for them [1].

The results of the study participants also confirm the influence of psychosocial environmental factors. In the risk group, coaching pressure and body comparisons with others are more pronounced—these mechanisms are considered key to the development of eating disorders in the literature. This risk is further increased by the fact that people in the risk group are less likely to perceive training as a tool for coping with stress and more likely to perceive it as a mechanism for weight and diet control—which corresponds to the psychoeducational need for intervention and psychological support. In the group of people exhibiting behaviours suggestive of eating disorders, a sense of pressure from the coach related to body image assessment was more frequently observed, suggesting a significant influence of external factors and environmental expectations on the formation of attitudes towards one’s own body (*p* = 0.005; V = 0.158). People at risk of eating disorders were significantly more likely to compare their appearance with that of their fellow trainees, confirming the intensified mechanisms of social comparison and critical self-assessment (*p* = 0.003; V = 0.164).

The strongest correlation was found in relation to the impact of regular physical activity on attitudes towards food. In the risk group, the vast majority of respondents (94.3%) indicated that training changed their approach to nutrition, while in the non-risk group this percentage was significantly lower (54%). This result may reflect a tendency to treat physical activity instrumentally as a tool for controlling food intake and body weight (*p* < 0.001; V = 0.404).

It is worth noting that the research group consisted of young athletes aged 17–20, i.e., individuals particularly vulnerable to pressure related to appearance and sports performance. Focusing on this specific population allows for a better understanding of the mechanisms shaping body image and the risk of eating disorders in the context of sports competition. An additional advantage is the large and balanced sample in terms of gender, as well as the use of standardised, reliable psychometric tools (EAT-26 and BES), which allow for comparison of results with other studies. The novelty of our study lies in the inclusion and detailed analysis of variables that have not previously been studied in the context of the risk of eating disorders (EDs) among physically active Polish adolescents. For the first time in the literature, the relationship between the risk of eating disorders (EDs) and pressure exerted by coaches, the role of physical activity, and young athletes comparing themselves to others has been presented. The study also included additional in-depth analyses, such as a linear regression analysis of ED risk in relation to the Body Esteem Scale score, which further reinforces the innovative nature of our work. Our results provide new insights into the role of these factors in shaping the risk of ED, making this study an important contribution to the scientific literature on the mental and physical health of young people. One limitation of the study is that it was based on self-reported survey data, which may lead to distortions–both from memory errors and deliberate manipulation of responses, especially with regard to sensitive issues such as eating disorders. The sample was limited to young people who actively participate in sports, which makes it impossible to generalise the results to the entire peer population. An additional drawback is the lack of objective physiological measurements that could provide more precise data on the health consequences of eating disorders, e.g., regarding muscle mass, body fat levels or the physical condition of athletes. Future studies should consider more in-depth measurements, such as body composition analysis using bioelectrical impedance analysis (BIA), which would allow for a better understanding of the issue and go beyond the limitations of the BMI alone.

## 5. Conclusions

The study demonstrated that a significant proportion of young athletes (17–20 years old) are at risk of developing eating disorders, with women showing a considerably higher vulnerability than men. The findings highlight the multidimensional nature of this risk, which is reflected in the imbalance between body image dimensions—individuals at risk tend to report lower self-esteem regarding attractiveness and weight control, while simultaneously rating their physical fitness highly. These results indicate that cultural and social pressures related to body ideals remain an important factor influencing young athletes, particularly women. The study also revealed a significant association between BMI and the risk of eating disorders, showing that underweight athletes are more prone to such disorders, while those with normal or higher body weight demonstrate lower susceptibility. This suggests that BMI and body composition should be carefully considered when assessing risk factors in sports environments.

Importantly, over 80% of at-risk athletes reported experiencing body-related pressure from coaches. This finding underscores the crucial role of the training environment in shaping athletes’ attitudes toward body image and eating behavior. Therefore, it is essential to incorporate educational and preventive interventions for coaches aimed at promoting body-positive communication and increasing awareness of their potential impact on athletes’ mental health.

The outcomes of this research emphasize the importance of early screening and preventive strategies for eating disorders in adolescent athletes. Healthcare providers, coaches, and educators should work together to raise awareness, monitor risk groups, and implement multidisciplinary support programs. Future studies should include objective physiological measurements and a more diverse sample to strengthen the generalizability of the findings and provide a more comprehensive understanding of the relationship between sports participation, body image, and eating disorders.

## Figures and Tables

**Table 1:** Characteristics of the group.

	Total (*n* = 428)	Women (*n* = 215)	Men (*n* = 213)	*p*-Value
Age [years] (X ± SD)	18.6 ± 1.13	18.7 ± 1.09	18.6 ± 1.19	0.561
Min–Max	17–20	17–20	17–20	
Height [cm] (X ± SD)	175 ± 9.92	168.7 ± 7.07	181.4 ± 8.14	<0.001 *
Min–Max	155–197	155–194	160–197	
Body mass [kg] (X ± SD)	68.5 ± 13.88	59.4 ± 10.03	77.6 ± 10.94	<0.001 *
Min–Max	40–115	40–90	52–115	
BMI [kg/m^2^] (X ± SD)	22.2 ± 2.99	20.9 ± 3.24	23.5 ± 2.04	<0.001 *
Min–Max	15.6–35.2	15.6–35.2	18.1–29.2	

X—average; SD—standard deviation. * *p* < 0.05.

**Table 2:** Distributions of participants across groups (total, women, men, at risk and without risk) for each studied variable (*n* = 428).

	Total (*n* = 428)		Female (*n* = 215)		Male (*n* = 213)	
	No Risk (*n* = 287)	Risk (*n* = 141)	No Risk (*n* = 117)	Risk (*n* = 98)	No Risk (*n* = 170)	Risk (*n* = 43)
Discipline *n* (%)						
Running	60 (20.9)	28 (19.9)	16 (13.7)	21 (21.4)	44 (25.9)	7 (16.3)
Hockey	37 (12.9)	3 (2.1)	2 (1.7)	1 (1)	35 (20.6)	2 (4.7)
Swimming	54 (18.8)	29 (20.6)	7 (6)	4 (4.1)	47 (27.6)	25 (58.1)
Volleyball	36 (12.5)	24 (17)	27 (23.1)	20 (20.4)	9 (5.3)	4 (9.3)
Gym	74 (25.8)	29 (20.6)	41 (35)	25 (25.5)	33 (19.4)	4 (9.3)
Dancing	26 (9.1)	28 (19.9)	24 (20.5)	27 (27.6)	2 (1.2)	1 (2.3)
*p*-Value	<0.001 *		0.382		0.002 *	
V-Cramer	0.323		0.157		0.299	
Does training help you cope with stress and life’s difficulties? *n* (%)						
Yes	194 (67.6)	77 (54.6)	77 (65.8)	54 (55.1)	117 (68.8)	23 (53.5)
Sometimes	83 (28.9)	40 (28.4)	33 (28.2)	29 (29.6)	50 (29.4)	11 (25.6)
Rarely	8 (2.8)	19 (13.5)	6 (5.1)	10 (10.2)	2 (1.2)	9 (20.9)
No	2 (0.7)	5 (3.5)	1 (0.9)	5 (5.1)	1 (0.6)	0
*p*-Value	<0.001 *		0.096		<0.001 *	
V-Cramer	0.238		0.172		0.360	
Do you feel pressure from your coach regarding the assessment of your physique? *n* (%)						
Yes	193 (67.2)	116 (82.3)	67 (57.3)	81 (82.7)	126 (74.1)	35 (81.4)
No	60 (20.9)	17 (12.1)	24 (20.5)	9 (9.2)	36 (21.2)	8 (18.6)
Sometimes	34 (11.8)	8 (5.7)	26 (22.2)	8 (8.2)	8 (4.7)	0
*p*-Value	0.005 *		<0.001 *		0.305	
V-Cramer	0.158		0.274		0.106	
Do you ever compare your appearance to the people you train with? *n* (%)						
Yes	178 (62)	110 (78)	76 (65)	85 (86.7)	102 (60)	25 (58.1)
No	69 (24)	22 (15.6)	14 (12)	8 (8.2)	55 (32.4)	14 (32.6)
Sometimes	40 (13.9)	9 (6.4)	27 (23.1)	5 (5.1)	13 (7.6)	4 (9.3)
*p*-Value	0.003 *		<0.001 *		0.933	
V-Cramer	0.164		0.270		0.25	
Does regular physical activity change your attitude towards food? *n* (%)						
Yes	155 (54)	133 (94.3)	73 (62.4)	93 (94.9)	82 (48.2)	40 (93)
No	132 (46)	8 (5.7)	44 (37.6)	5 (5.1)	88 (51.8)	3 (7)
*p*-Value	<0.001 *		<0.001 *		<0.001 *	
V-Cramer	0.404		0.386		0.363	

* *p* < 0.05; *p*-values were calculated using Chi-square tests for categorical variables. V-Cramer indicates effect size.

**Table 3:** Results of ANCOVA for EAT-26 Scores by Type of Physical Activity, Controlling for Gender, Age, and BMI.

Factor	F	*p*
Discipline	1.074	0.374
BMI	0.024	0.878
Age	0.223	0.637
Gender	12.448	<0.001 *
Interaction: Type of Physical Activity ✻ Gender	0.885	0.491

* *p* < 0.05; *p*-values were calculated using ANCOVA; ✻—interaction term.

**Table 4:** Distribution of participants according to risk of eating disorders (EAT-26) and gender, BMI and body esteem scale (BES) (*n* = 428).

EAT-26	Total *n* = 428	No Risk *n* = 287	Risk *n* = 114	*p*-Value	V-Cramer
Gender *n* (%)					
Female	215 (50.2)	117 (40.8)	98 (69.5)	<0.001 *	0.27
Male	213 (49.8)	170 (59.2)	43 (30.5)		
Body Mass Index *n* (%)					
Underweight	47 (11)	21 (7.3)	26 (18.4)	<0.001 *	0.212
Normal	326 (76.2)	227(79.1)	99 (70.2)		
Overweight	52 (12.1)	39 (13.9)	13 (9.2)		
Obesity	3 (0.7)	0 (0)	3 (2.1)		
Physical Attractiveness *n* (%)					
Low	176 (41.4)	97 (33.8)	79 (56)	<0.001 *	0.231
Medium	135 (31.5)	95 (33.1)	40 (28.4)		
Hight	117 (27.3)	95 (33.1)	22 (15.6)		
Weight Control *n* (%)					
Low	122 (28.5)	55 (19.2)	67 (47.5)	<0.001 *	0.322
Medium	153 (35.7)	105 (36.6)	48 (34)		
Hight	153 (35.7)	127 (44.3)	26 (18.4)		
Physical Condition *n* (%)					
Low	145 (33.9)	123 (42.9)	22 (15.6)	<0.001 *	0.291
Medium	135 (31.5)	87 (30.3)	48 (34)		
Hight	148 (34.6)	77 (26.8)	71 (50.4)		

* *p* < 0.05; *p*-values were calculated using Chi-square tests for categorical variables. V-Cramer indicates effect size.

**Table 5:** Means and standard deviations of EAT-26 and BES scores by gender (*n* = 428).

Variable	Total (*n* = 428) (X ± SD)	Female (*n* = 215) (X ± SD)	Male (*n* = 213) (X ± SD)	*p*-Value	Effect Size
EAT-26 Total	15.84 ± 12.94	20.33 ± 13.64	11.31 ± 10.42	<0.001 *	0.744
Dieting	9.52 ± 7.82	11.82 ± 8.69	7.19 ± 6.01	<0.001 *	0.619
Bulimia	2.16 ± 3.11	3.03 ± 3.57	1.28 ± 2.25	<0.001 *	0.588
Oral Control	2.49 ± 3.23	3.48 ± 3.56	1.49 ± 2.51	<0.001 *	0.645
Body Esteem Image					
Physical Attractiveness	40.07 ± 8.18	39.36 ± 8.39	40.78 ± 7.90	0.072	−0.174
Weight Control	31.17 ± 10.13	26.76 ± 10.02	35.61 ± 8.11	<0.001 *	−0.970
Physical Condition	40.24 ± 14.26	30.51 ± 10.02	50.07 ± 10.72	<0.001 *	0.787

* *p* < 0.05; X—average; SD—standard deviation; *p*-values were calculated using Mann–Whitney U tests for continuous variables. Cliff’s delta indicates the effect size.

**Table 6:** Linear regression analysis of EAT-26 and BES subscale score in sex group (*n* = 428).

Model Coefficients–EAT-26; R = 0.551 R^2^ = 0.304				
Predictor	β	SE	t	*p*-Value
Body Esteem Scale				
BES–Physical Attractiveness	0.063	0.092	0.69	0.494
BES–Weight Control	−0.462	0.089	−5.15	<0.001 *
BES–Physical Condition	−0.057	0.080	−0.72	0.237
Gender				
Male–Female	−3.626	1.707	−2.12	0.034 *
Body Mass Index				
Normal–Overweight	−3.824	1.670	−2.289	0.023 *
Underweight–Overweight	1.202	2.486	0.483	0.629
Obesity–Overweight	22.441	6.651	3.374	<0.001 *
Physical Activity				
Hockey vs. Running	−2.052	2.208	−0.929	0.353
Swimming vs. Running	3.360	1.731	1.941	<0.05 *
Volleyball vs. Running	−0.298	1.974	−0.151	0.880
Gym vs. Running	0.732	1.667	0.439	0.661
Dance vs. Running	−0.606	2.043	−0.297	0.767

Reference category for gender is “male”; EAT-26—Eating Attitudes Test-26; SE— standard error; t—t-statistics; β—regression coefficients; * *p* < 0.05.

## Data Availability

The raw data supporting the conclusions of this article will be made available by the authors on request.
